# Strategic Focus on 3R Principles Reveals Major Reductions in the Use of Animals in Pharmaceutical Toxicity Testing

**DOI:** 10.1371/journal.pone.0101638

**Published:** 2014-07-23

**Authors:** Elin Törnqvist, Anita Annas, Britta Granath, Elisabeth Jalkesten, Ian Cotgreave, Mattias Öberg

**Affiliations:** 1 Institute of Environmental Medicine, Karolinska Institutet, Stockholm, Sweden; 2 Former AstraZeneca R&D, Södertälje, Sweden; 3 Institute of Biomedicine, The Sahlgrenska Academy, University of Gothenburg, Gothenburg, Sweden; 4 Karolinska University Hospital, Stockholm, Sweden; 5 Swedish Toxicology Sciences Research Center, Södertälje, Sweden; Medical University of Graz, Austria

## Abstract

The principles of the 3Rs, Replacement, Reduction and Refinement, are being increasingly incorporated into legislations, guidelines and practice of animal experiments in order to safeguard animal welfare. In the present study we have studied the systematic application of 3R principles to toxicological research in the pharmaceutical industry, with particular focus on achieving reductions in animal numbers used in regulatory and investigatory *in vivo* studies. The work also details major factors influencing these reductions including the conception of ideas, cross-departmental working and acceptance into the work process. Data from 36 reduction projects were collected retrospectively from work between 2006 and 2010. Substantial reduction in animal use was achieved by different strategies, including improved study design, method development and project coordination. Major animal savings were shown in both regulatory and investigative safety studies. If a similar (i.e. 53%) reduction had been achieved simultaneously within the twelve largest pharmaceutical companies, the equivalent reduction world-wide would be about 150,000 rats annually. The results point at the importance of a strong 3R culture, with scientific engagement, collaboration and a responsive management being vital components. A strong commitment in leadership for the 3R is recommended to be translated into cross-department and inter-profession involvement in projects for innovation, validation and implementation. Synergies between all the three Rs are observed and conclude that *in silico-, in vitro-* and *in vivo-*methods all hold the potential for applying the reduction R and should be consequently coordinated at a strategic level.

## Introduction

The 3Rs, defined as Replacement, Reduction and Refinement, are fundamental principles for driving ethical research, testing and education using animals. The principles were proposed by Russel and Burch in 1959 [1], and since have been widely accepted and adapted to modern society in general and to science research in particular, as described at the Sheringham workshop 1995 [2] and in Bologna 1999 [3]. The 3Rs are currently incorporated as a key concept for humane use of animals in research into various important legislations, for instance in the European Union (EU) [4], Brazil [5] and Japan [6]. They are also implicit in the respective Animal Welfare Acts in the United States [7], in China [8] and in India [9], [10]. The principles of the 3Rs are also well integrated into international guidelines for toxicity testing, such as the guidelines developed by the Organization for Economic Co-operation and Development [11], and into regulatory test guidelines applicable for safety assessments/toxicity evaluations for chemicals [12], [13], pesticides [14], cosmetics [15] and pharmaceuticals [16]. National 3R advisory boards, or 3R centers, have been active in Canada since 1968 [17], in Australia and New Zealand since 1987 [18] and in UK since 2004 [19], each tasked with funding 3R research, performing surveys on views in the area of animal ethics and promoting an open dialogue about research on animals. The newly adopted EU directive on the protection of animals used for scientific purposes demands of members of the Union to increase collaboration in the 3Rs, which puts a pressure on the member states to establish national 3R centers [4]. Several 3R centers have been recently started, such as FICAM in Finland in 2004 [20], and the Netherlands Knowledge Centre on Alternatives to animal use (NKCA) in 2010 [21].

Although officially embraced in various legislations and guidelines, and despite strong private and official initiatives, the principles of the 3Rs are not yet fully incorporated into the everyday animal-based research. The discrepancy between the 3Rs vision and general, everyday practices at both regulatory authorities and research units in the industry or academia is well illustrated in public surveys. In a survey of Canadian researchers' views on the 3Rs, the respondents will to embrace the 3Rs was recorded, but there was a clear lack of knowledge in how to transform the 3R principles into practice [22]. Further, the responding researchers did not see replacement of animals in research as achievable or realistic. There was also an attitude that the 3Rs not only would disturb the research process in terms of time and cost, but would also compromise research data, primarily due to loss of statistical significance when applying the reduction R. The need for increased efforts and plans for continuous education in animal ethics is pointed out in a survey of members of the Canadian Institutional Animal Care and Use Committees [23]. In “Views on the 3Rs”, a survey report from 2012 by the Department for Business, Innovation and Skills in the United Kingdom, scientists and animal care staff were asked when during the research process the 3Rs was considered [24]. As many as 95% of the respondents considered the 3Rs when commencing their projects, dropping to 50% during execution of the work to about 25% when presenting data at conferences or meetings. This trend clearly shows that opportunities for cooperation and sharing information and best practice are not being fully exploited. In addition, an increase of animal use in research in general, and a shortcoming in the efforts to implement the 3Rs, especially reduction, is illustrated by the increase in the percentage of articles reporting animal use in four high ranking journals between the years of 1983 and 2007 [25].

Today there is no globally standardized way of reporting on any of the 3R principles, and legislation and reporting of animal use in relation to the 3Rs varies strongly between different countries. This presents difficulties in obtaining an overview of the progress and implementations of the 3Rs regionally, nationally and globally. In advance of this, visions and measurable goals for driving 3R-based improvements in biomedical research differ between both individual countries and different areas of research, and there is urgent need for a more structural and effective exchange of method developments, databases and best-practice examples. There is also a need to separate the 3Rs in order to assimilate academic and practical expertise for each R individually [26], as well as a need for greater academic focus on the 3R research, in order to increase the status of the research area [27].

According to European commission data from 2011, about 11.5 million laboratory animals were used annually in the European Union, and 8.75% of these animals were used for toxicological evaluations and safety assessments, with every second animal used to fulfill legislative demands [28]. Many 3R initiatives have been developed in toxicological research, both at the research and legislative levels. One concerted paradigm is in the field of toxicity testing of cosmetics, where the 3R vision was imprinted by implementation of the regulation on cosmetic products, enhancing and supporting the continuous work with establishment and validation of alternative methods [15]. More than 80 methods have been validated, of which 50 are *in vitro* tests, 10 use isolated organs and the remainder are refined *in vivo* methods regarded as more humane to the animals [29]. A recent example of a research initiative within the field of testing of cosmetics is the “Safety Evaluation Ultimately Replacing Animal Testing” (SEURAT-1) that cluster the research efforts of over 70 European universities, public research institutes and companies and focuses on the complex area of repeated dose toxicity [30]. These efforts in toxicology may present great opportunities for cross-fertilizing efforts into other areas of biomedical research, where 60% of the animals utilized in the EU find application, and where little or no concerted effort is being applied to develop 3R-based research paradigms.

In contrast to these efforts in the public research forum, little information is available from application of 3R principles within industrial research. In the present study we display various methods to implement 3R thinking into the core operating practices of a multi-national pharmaceutical company, from the perspective of toxicity testing for human risk assessment. Data was obtained from the Safety Assessment Unit at AstraZeneca R&D, Sweden, whose operating model was focused on performing preclinical regulatory and investigative toxicity testing of future medicines. In a retrospective review, animal use data from fully implemented 3R projects were collected over a 5 year implementation period from 2006 to 2010, and the analysis combined with detailed mapping of how, where and why these 3R ideas occurred and how they were implemented. The overall aim of the present study was to explore strategies for future ways of working with the 3Rs locally and globally within a research organization performing investigative and regulatory toxicity studies. In addition, the aim was to study the importance of involvement and empowerment of all employees at different levels of the organization to consider the 3Rs in their everyday work. The results reveal achievement of considerable reductions in animal uses, and the major factors which dictate this in a large commercial organization. The results are also discussed in terms of propagation of best-practice to other organizations in order to maximize impact on animal welfare.

## Materials and Methods

**Figure 1:**
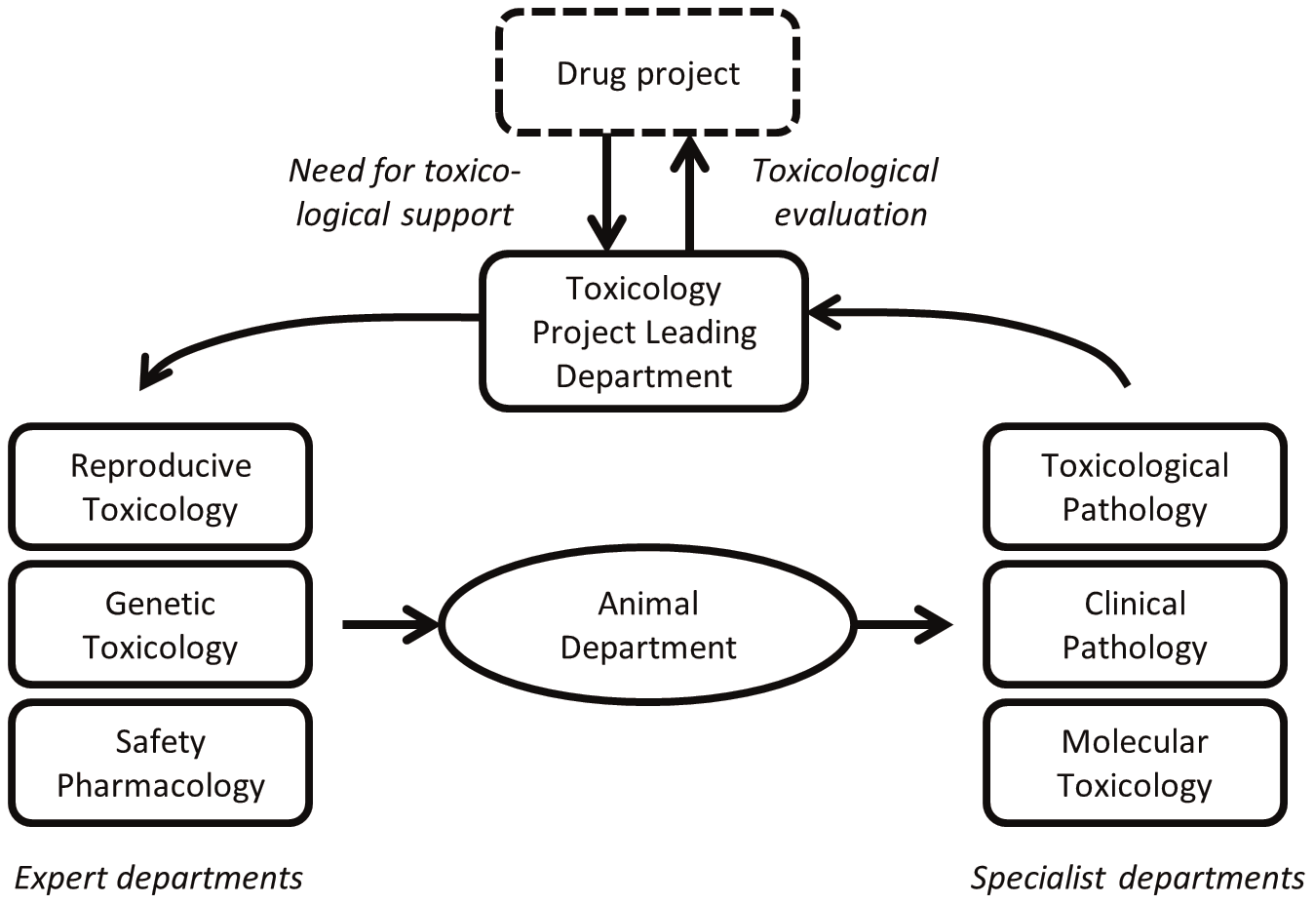
Schematic illustration of the Safety Assessment Research Unit at AstraZeneca, Södertälje. The unit comprised eight departments and performed preclinical toxicity studies and evaluations to support the company's various drug projects to predict potential side effects before e.g. clinical trials in man.

All data were obtained from the Safety Assessment Research Unit at AstraZeneca in Södertälje, Sweden. At the time of this retrospective study the unit comprised of eight departments (Figure 1), including 300 staff (scientists, technicians, veterinarians, quality assurance experts and managers). The research unit performed preclinical toxicity studies and evaluations according to regulatory guidelines, as well as carrying out studies on investigative basis, in order to support the company's various drug projects in predicting potential side effects before clinical trials in man. During 2009, the unit completed over 300 scientific preclinical safety assessment reports of which one third was performed *in vitro* and two thirds *in vivo*. Test species for in vivo studies included the rat, mouse, rabbit and dog. All animals were acclimatized before start of study and test substances were administered orally, intravenously, subcutaneously or via inhalation depending on anticipated future route of administration in man. Toxicological evaluations were based on *in vivo* findings such as blood and urine samples, and on toxicological findings at necropsy. Humane endpoints were decided for each study as well as method of sacrifice appropriate for the species used. All species were included in the systematic work with 3R described below. All *in vivo* research was performed in a Good Laboratory Practice (GLP) facility in accordance with national legislations for research on animals and the EU directive on the protection of animals used for scientific purposes (86/609/EEC), and was ethically approved by an external ethical committee (the Ethical Committee of Animal Experiments in Stockholm). Lean Sigma process optimization was also operative throughout the test period. The unit was closed by December 2012.

In order to review the outcome of different 3R projects aimed at reduction, all reduction improvements implemented from 2006 to 2010 at the Safety Assessment Research Unit were collected retrospectively. Reduction was defined as *”any methods for obtaining comparable levels of information from the use of fewer animals, or for obtaining more information from the same number of animals”.* This definition is in accordance with the widely accepted conclusions and recommendations of the Sheringham workshop from 1995 [2]. Each reduction project included had been thoroughly discussed and validated before management approval and implementation.

All collected reduction improvements implemented were categorized according to three different categories:

(1) “Improved study design”, defined as projects resulting in a reduction of animal use either after decreasing the number of animals in test groups or excluding test groups in a certain study type, or by excluding certain parameters/study types in series of studies. The decisions to change the study design were based on experience, historical data or increased knowledge, and consequently resulted in a scientifically more optimized study design as well as a reduction of animal use.

(2) “Method development”, defined as projects resulting in a reduction of animal use achieved by introduction of more sensitive assays/techniques, facilitating smaller amounts of body fluids or tissues for analyses and optimizing delivery of test parameters from the same animal instead of using individual animals for each parameter. This results both in more scientific information per animal and in a reduction of number of animals needed in each test group. Other methods developed to reduce the number of animals used were *in vitro* assays for screening to exclude compounds before testing *in vivo*, as well as *in silico* methods enabling more effective processing of research data.

(3) “Coordination”, defined as projects resulting in a reduction of animal use achieved by increased collaboration and communication between various contributing departments. This enabled combining different scientific aims from separate areas of toxicology testing in one study, and the sharing of control animals when applicable. Reduction by coordination was also achieved by combining *in vivo* and *in vitro* studies instead of using separate animals for *in vitro/ex vivo* testing, as well as by biobanking samples for future use. Combining such studies could be planned in advance by increased communication and collaboration between departments.

Before and after the implementation of the new procedure, the number of animals used (per study and/or per year) was estimated by the project owner for each reduction project. Collated data per year were derived and a measure of overall reduction per year based on animal use and number of toxicity studies during 2009 for each species. For some projects the actual reduction in numbers was imprecise in nature due either to irregular use of the study, or to the technique described, or in the extent the future need and use of a certain technique/process, particularly in the case of the preclinical biobank. The overall reduction is therefore likely to be slightly underestimated.

Each reduction project was linked to departmental ownership of the project in order to map and analyze involvement and engagement into the 3R work at the Safety Assessment Research Unit. In cases where project ownership was shared between two or more departments, all departments involved were recorded. General 3R-projects aiming at high-level organization of different parts of the 3R work at the unit were also listed.

## Results

A total of 36 implemented reduction projects were identified for the period 2006 to 2010. Of these, 32 were implemented on rat studies (Tables 1–3) and four were dog-specific (not included in tables). Several of the identified rat-projects were also applicable for other species, i.e. mice, rabbits and dogs.

**Table 1:** Projects related to improvements in study design, the reduction per study and estimated annual reduction.

No.	Project description	Number of studies in 2009	Reduction per study	Annual reduction
1.	***Dose-range finding studies in reproduction toxicity studies.*** Birth groups excluded from standard design and only included if birth issues are indicated. Reduction from 66 to 48 females and from 180 to 0 puppies per study.	3	246 to 48	594
			(80%)	
2.	***Combining studies of embryo-fetal development and female fertility.*** Evaluation in one combined study instead of performing two separate studies by adjusting dosing, mating and necropsy to fit for both scientific purposes.	2	160 to 80	160
			(50%)	
3.	***Combining the micronucleus test and the comet assay*** by adjusting dosing and necropsy to fit for both scientific purposes. Reduction from 63 rats (28 rats in the micronucleus test, and 45 rats in the comet assay) to 32 rats per combined study.	3	63 to 32	93
			(49%)	
4.	***Optimized project design for inhalation projects***. Based on experience and scientific evaluation of inhalation studies, it was concluded that the results from the 1-month study could be dose-setting for the 6-month study, hence the 3-month study could be excluded.	NA	354 to 236	a)
			(33%)	
5.	***New dosing routine for oral micronucleus tests***. Evaluation after single dosing was excluded and two dose regimen was adopted as standard, enabling detection of micronucleus developed within 24 and/or 48 hours after dosing in the same animal.	10	42 to 28	140
			(33%)	
6.	***New dosing routine for intraveneous micronucleus tests.*** Evaluation after single dosing was excluded and two dose regimen was adopted as standard, enabling detection of micronucleus developed within 24 and/or 48 hours after dosing in the same animal.	10	42 to 28	140
			(33%)	
				
7.	***Fewer animals in the embryo-fetal development studies.*** Change of group size from 22 to 20 females per group due to documented high pregnancy rate at the facility. Reduction from 88 to 80 rats per study. (Also applicable for rabbits)	3	88 to 80	24
			(9%)	
8.	***Optimized control groups in investigative pathology toxicity studies.*** Control groups are reduced by 50% by using historical data with background variation.	NA	b)	b)
				
9.	***Shared control groups in investigative studies.*** The same control group was used for more than one scientific issue when scientifically motivated. (Applicable for any species)	NA	b)	b)
				
	***Total Estimated Annual Animal Reduction***			*1 151*

NA = Not analysed

a) Not possible to estimate as the routine was implemented just before the end of the investigated period.

b) Not possible to estimate as the design and number of investigative studies varies a lot between years. To be evaluated after 2–3 years.

**Table 2:** Projects related to development of new methods, the reduction per study and estimated annual reduction.

No.	Project description	Number of studies 2009	Reduction per study	Annual rat reduction
10.	***New computer program for safety pharmacology parameters in dogs.*** The program enabled inclusion of respiratory tests into telemetry studies in dogs. Hence, separate respiratory studies in rats could be excluded.	4	40 to 0	160
			(100%)	
11.	***New in vitro assays based on in vivo results.*** Screening proposed drug candidates without *in vivo* studies in drug projects with anticipated side effects such as gallbladder or hepatic toxicity. (applicable to all species)	3	60 to 0	180
			(100%)	
12.	***Combining 3 pathological examinations.*** Using one rat instead of three in a 3D-reconstruction of the heart.	NA	3 to 1	a)
			(67%)	
13.	***New blood sampling routines in inhalation toxicity studies***. Previously, blood sampling for toxicokinetics was performed via heart puncture at euthanasia as large blood volumes were needed for analytical determination. New analytical methods enabled lower blood levels (500 µL) and repeated sampling via the tail vein.	15	280–340 to 136	2160–3060
			(51–60%)	
14.	***Micro sampling in dose-range finding studies for further reproduction toxicity testing.*** No extra groups for toxicokinetics evaluation	3	48 to 24	72
			(50%)	
15.	***Micro sampling in the test for physical dependence, safety pharmacology.*** No extra groups with 4–8 animals for toxicokinetic evaluation. Reduction from 48–64 to 32 animals per study.^d^	3	48–64 to 32	48–96
			(33–50%)	
16.	***New method for blood sampling for coagulation evaluations***. Sampling via tongue vein instead of heart puncture. Extra group of 40 animals for coagulation could be excluded.	NA	140–158 to 100–118	a)
			29–34%	
17.	***Micro sampling for regulatory toxicity studies with oral, subcutaneous or intravenous exposure.*** Reduced blood volumes needed for kinetic evaluations in studies longer than 14 days. Reduced number of extra animals. Reduction from 136 to 100 rats per study. (applicable also in mice)	15	136 to 100	540
			(26%)	
18.	***New blood sampling routines in inhalation toxicity studies***. Further development of project No. 13, a new analysis method for kinetics enabled even lower sample volume (from 500 µL to 300 µL) and consequently reduced number of extra animals for blood sampling.	15	136 to 118	270
			(13%)	
19.	***New in vitro screening for testing*** of *bone marrow toxicity*. *In vitro* screening in three steps: if tested positive in *in vitro* mouse cell line test, the substance is tested *ex vivo* in human cell culture. If still positive, tested on bone marrow from animals euthanized after exposure for the substance. Reduction as a result of fewer drug candidates to be tested *in vivo*.	NA	a)	a)
20.	***New in vitro model for liver toxicity***. A bioreactor that simulates human liver function aiming at better simulation of the human physiology and at avoiding use of primary animal liver cells.	NA	b)	b)
21.	***Continuously discovering new biomarkers for safety assessments***. Results in better diagnostics, more information, more proper decisions and evaluations which may result in minimized group sizes and optimized study design and early decisions of toxicity and risk of side effects in humans and by that avoiding “unnecessary” research in animals.	NA	c)	c)
	***Total estimated annual animal reduction***			3430–4378

NA = Not analysed

a) Not possible to estimate as the test/study is performed when requested and this varies substantially between years.

b) Not possible to estimate the effects on reduction of animal use or replacement of animal studies today.

c) Not possible to estimate as the number of biomarker studies and study design varies depending on present drug projects and safety assessment issues. Probably a reduction of more than one hundred animals per year. To be evaluated after 2–3 years.

d) Possible risk for interference with observations of clinical signs is addressed and during scientifically investigation and evaluation before implementing the method as default.

**Table 3:** Projects related to coordination, the reduction per study and estimated annual reduction.

No.	Project description	Number of studies in 2009	Reduction per study	Annual rat reduction
22.	***Combining micronucleus studies and regulatory 1-month toxicity or shorter early high dose studies***. Bone marrow sampling at necropsy when applicable. Reduction of animal use in separate micronucleus studies (28 animals per study).^f^	NA	28 to 0	a)
			(100%)	
23.	***Coordination of ordering of animals.*** Better communication and more homogenous size of delivered animals reduced the number of animals from 10 to 5% extra. Reduction from 12 to 6 per study. (also applicable for mice)	35	12 to 6	210
			(50%)	
24.	***Combining 3 scientific evaluations in lung pathology examinations***. BAL, organ weight, and histopathology were examined in one instead of two animals.	NA	64–24 to 32–12	a)
			(50%)	
25.	***Combining three scientific evaluations in investigative studies in safety pharmacology***.	1	168 to 102	66
			(39%)	
26.	***Including male fertility testing in the 6 months regulatory toxicity study.*** 90 rats in the six months study and 160 rats in male fertility studies were reduced to 90 rats plus 80 females to be mated.	2	250 to 170	160
			(32%)	
27.	***Combining behavior/physiological tests and inhalation toxicity tests***. Including Irwin tests in inhalation studies. Separate inhalation: 98 rats per inhalation study and 36 separate rats for Irwin test were reduced to 118 per combined study.	6	154 to 118	216
			(23%)	
28.	***Using the same vehicle control.*** The vehicle control was used for several test substances when applicable.	NA	a)	a)
29.	***Setup of a Preclinical Biobank***. Archive samples collected from all studies to avoid repeated *in vivo* studies for possible future need of additional/completing information such as biomarkers. (applicable for all species)	NA	b)	b)
30.	***Setup of a Biobank with control samples.*** A biobank for future use at method development or validation. (applicable for all apecies)	NA	c)	c)
31.	***Including biomarker studies in regulatory toxicity.*** By receiving tissue and fluids from animals in ordinary test groups when applicable. (applicable for all species)	NA	d)	d)
32.	***New routines for thorough discussions.*** Scientist from different fields/departments discuss before start of study in order to maximize the scientific value and reduce unnecessary use of animals. (applicable for all species)	NA	e)	e)
	***Total estimated annual animal reduction***			652

NA = Not analysed

a) Not possible to estimate as the number of studies varies between years. To be evaluated after 2–3 years.

b) Not possible to estimate as the number of investigative studies varies between years. Probably an annual reduction of more than hundred rats. To be evaluated after 2–3 years.

c) Not possible to estimate as the number of method development and/or validation studies varies between years. Probably an annual reduction of more than one hundred rats. To be evaluated after 2–3 years.

d) Not possible to estimate as the number of biomarker studies varies depending on present drug projects and safety assessment issues. Probably an annual reduction of more than one hundred rats. To be evaluated after 2–3 years.

e) Not possible to estimate as the projects vary in nature.

f) Risk for too low exposure for micronucleus evaluation is addressed and during scientifically investigation and validation before implementation as default.

Projects were related to either improvement of study design, method development or coordination. The improved study designs (Table 1) resulted in a total estimated annual animal reduction of 1151 rats, which equates to 20% of the total estimated reduction. Half of this reduction was achieved by excluding birth groups from dose-range finding studies in reproduction toxicity studies (Project No. 1). The introduction of new dosing-routines (No. 5 and 6) was the second most important reduction strategy, reducing the estimated number of rats by 280 annually in oral and intravenous micronucleus tests. Improving the study design so that two separate studies could be merged into one (No. 2 and 3) was the third most important strategy, reducing the annual use of rats by 253. Dose-setting for 6-months inhalation toxicity studies were previously based on a 3-month study. An evaluation showed that this study could be excluded and the dose-setting based on a 1-month study, reducing the number of animals with 354 to 236 (No. 4). The annual impact of this study design improvement was not assessed since the project was implemented at the very end of the investigated period. In one project (No. 7) the number of animals was reduced due to smaller group sizes, a result of high pregnancy rates at the facility. Finally, two projects (No. 8 and 9) reduced the number of animals by introducing historical data for control and shared control groups. It was, however, not possible to estimate the annual impact of all these projects within the time-frame of the evaluation.

Method developments had a substantial impact on reduction of laboratory animals and resulted in an estimated reduction by 3430–4378 rats, more than two thirds of the total estimated annual animal reductions achieved at the unit over the test period (Table 2). Development of computer programs (Project no. 10) and new *in vitro* methods (No. 11, 19 and 20) resulted in 67–100% reduction in use of animals, corresponding to a reduction of at least 340 rats every year. The most important method improvements, in terms of reduced number of animals, were the development of new blood sampling techniques. For example, micro sampling enabled fewer satellite animals used for toxicokinetic evaluations. When this and other new blood sampling routines were introduced, the estimated use of rats dropped by approximately 3500 rats annually. A minor reduction was also achieved by new pathological methods (No. 12). Reduction projects involving continuous development of methods for biomarkers (No. 21) were not quantifiable in terms of number of animals, but indicate a movement forward with future possibilities for both reduction and replacement of animal use.

Eleven reduction projects were related to coordination of the work with laboratory animals (Table 3). Annual reduction in use of rats achieved by coordination was estimated to 652 animals, which equals 11% of the total estimated reduction at the safety assessment research unit. Five of the projects (Projects No. 22 and 24–27) were achieved by coordination between different research areas at the Safety Assessment Research Unit, which resulted in increased scientific data from each animal used. Coordinated purchase and ordering of animals (No. 23) resulted in a reduction in use of “extra” rats by 50%. Several reduction projects in the category of coordination were not possible to quantitatively evaluate, including the coordination of controls with the same vehicle (No. 28); establishment of a preclinical biobank (No. 29); biobank for control animal tissues (No. 30), studies of biomarkers in regulatory toxicity testing to avoid separate *in vivo* studies (No. 31); and introduction of routines for thorough interdepartmental discussions to avoid unnecessary use of laboratory animals (No. 32).

**Figure 2:**
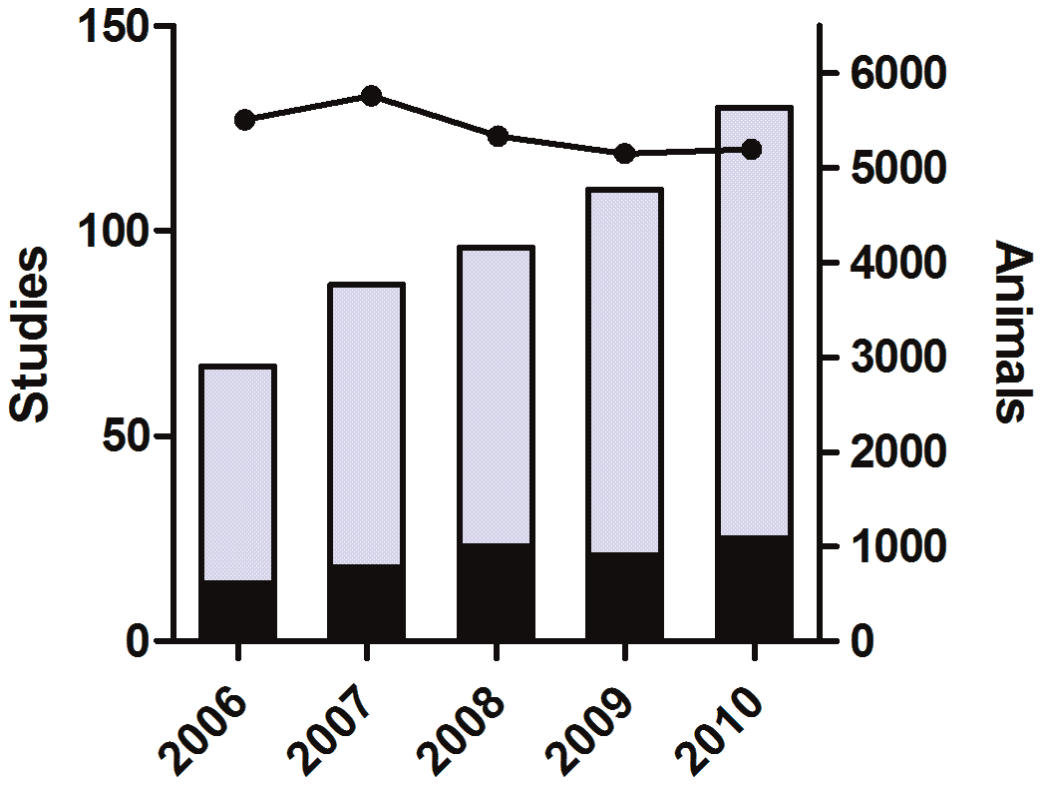
Total number of rats used in toxicity studies at the Safety Assessment Research Unit (black line) and total number of reported regulatory toxicity studies, one month or longer (black bars) and shorter investigative toxicity/mechanistic studies (grey bars) during the years 2006–2010.

Overall, as a result of the 32 implemented reduction projects the unit was able to increase the number of regulatory toxicity studies, mainly with long-term exposure, with 79%, from 14 studies in 2006 to 25 studies in 2010 (Figure 2). An even higher increase was found for investigative studies. This type of shorter duration studies almost doubled, from 53 studies in 2006 to 105 studies in 2010.

**Figure 3:**
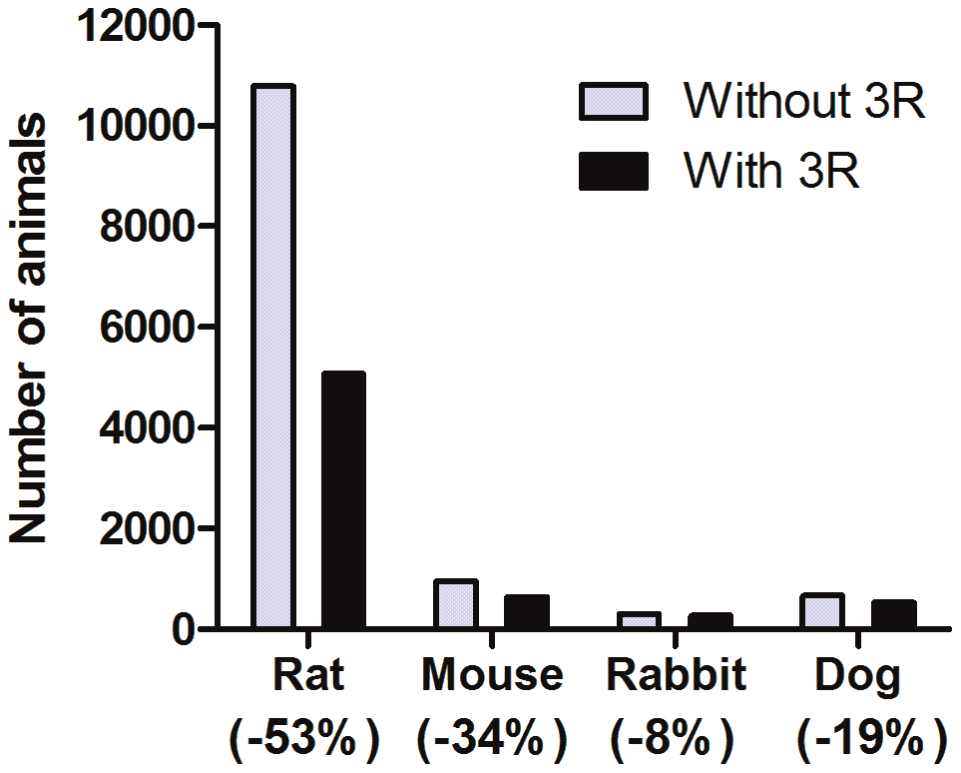
Estimated reduction in number of animals used in toxicity studies at Safety Assessment Research Unit. Actual number of animals used in 2009 (black bars) compared with the estimated number of animals if no reduction projects would have been implemented (grey bars).

Several of the identified reduction rat-projects were also implemented for the other species used at the unit, with significant reduction of the number of animals as a result (indicated in the footnote of tables 1–3); mice (9 projects); rabbits (7 projects); and dogs (6 projects). Moreover, four projects were implemented only for dogs, including two projects of optimized study design and two projects with new routines related to telemetry during safety pharmacology studies, reducing the annual use of dogs with 130. Based on all studies performed at the unit 2009, the actual number of animals was compared with the number of animals that would have been used per study if none of the reduction projects would have been performed. The implemented reduction projects resulted in an estimated reduction (only including projects with quantifiable reduction) of animal use in all species ranging from 8% in rabbits to 53% in rats (Figure 3). Assuming no reduction strategy implemented, the actual number of animals would have been significantly higher; +5707 rats, +324 mice, +24 rabbits and +130 dogs, a total saving of over 6000 animals every year.

**Figure 4:**
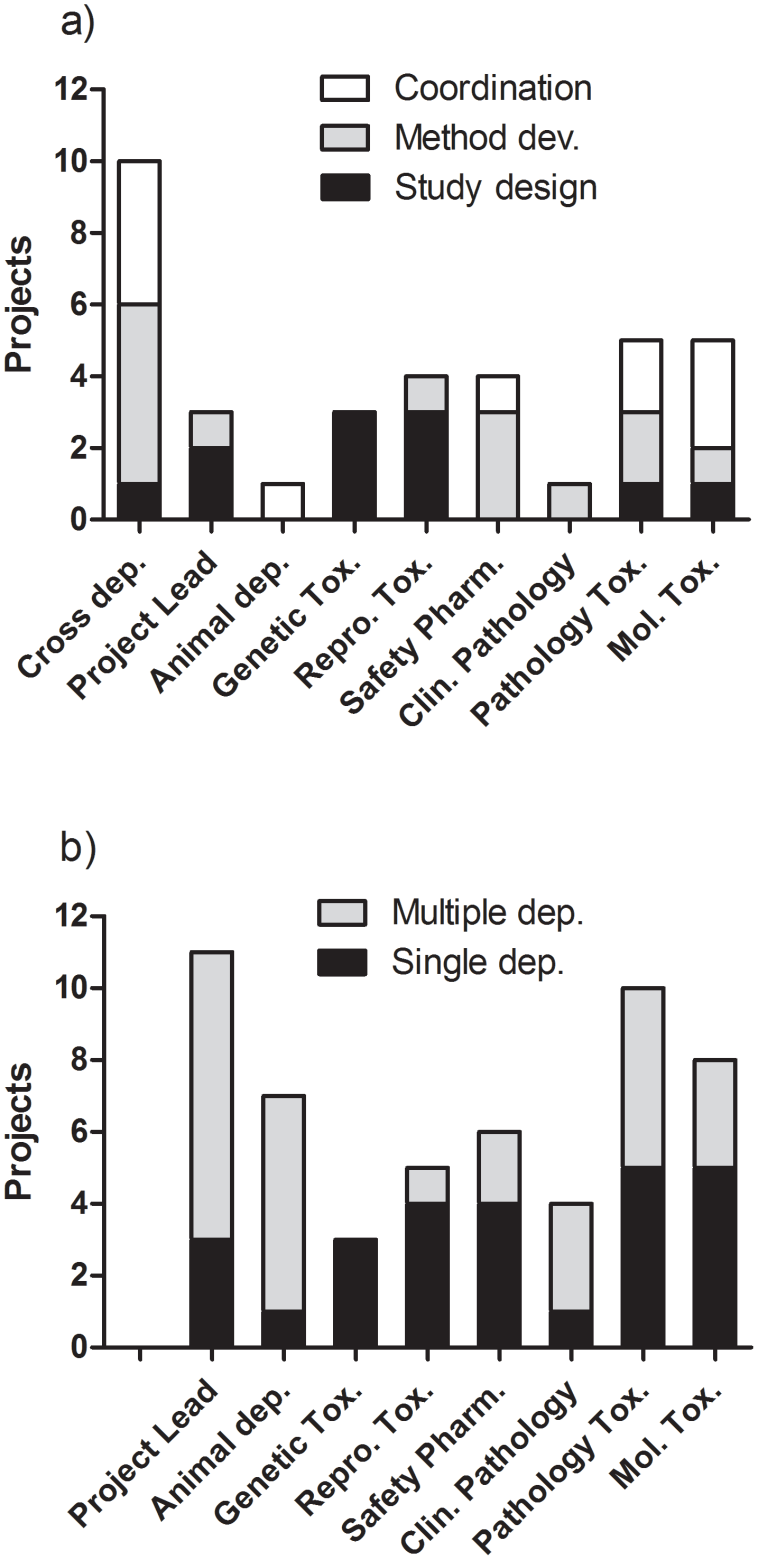
Projects linked to the department ownership (a) or the participation (b) of each project. The number of projects specified according to category of the project (a) and specified in relation to the participation in projects with multiple or single departments (b).

The organizational analysis showed that initiative and implementation of the reduction projects was organized from all eight departments at the unit (Figure 4a). The three different reduction categories were scattered over the different departments (Figure 4a). One of eleven study design improvements were initiated cross departments, whereas a larger proportion of projects related to method development (5/14) and coordination (4/11) were initiated cross-departmentally. Single department initiatives came both from the expert departments upstream of the animal department (11 projects) and the specialist departments downstream of the animal department (11 projects). Few single department initiatives were initiated from the project leader and the animal department. However, one or both of these two departments were involved in nine of the ten cross-departmental projects (Figure 4b). One fourth (10/36) of the reduction projects was initiated by two or more of the departments at the unit (Figure 4a).

## Discussion

### Three strategies for reduction

By detailed polling and analysis of a retrospective survey over a five year period and by collating projects which achieved reductions in animal use either by (1) improved study design, (2) method development or (3) coordination, each of the three categories were shown to contribute significantly to the overall reductions achieved. We identified 36 reduction projects, of which 32 were implemented on the use of rats (Tables 1–3). Several of the rat-projects were also implemented on the use of other test species (mice, rabbits and dogs), whereas four projects were only implemented on dogs (data not shown). All three strategies were equally common. However, in terms of differential effects on reductions in the use of rats, 20% was due to improved study design, 68% of the total reduction was achieved due to method development and 12% due to new projects related to tactical coordination.

A main strategy to achieve reduction of the number of animals was through modification of the study design (Table 1). When implemented in studies with large number of animals, such as the reproduction toxicity studies, the impact was shown to be substantial. Reductions related to study design were also achieved by adjusting schedules for dosing, mating and necropsy, to fit the purposes of two separate studies in to one study. The number of control animals was also reduced by using historical data or by using the same control group for several investigative issues. Without a systematic organizational structure for 3R including all departments, as discussed below, this type of coordinated design shift would have been impossible. Moreover, a minor reduction was achieved by the change of group size in embryo-fetal development studies due to documented high pregnancy rate at the facility. The latter project is a strong example of a synergy between the two Rs for refinement and reduction.

Method development was shown to be the most efficient strategy for achievement of reduction in animal use (Table 2). One of the most influential improvements was the introduction of new blood micro-sampling methods. This innovative method for reduction was based on previous improvements in analytical abilities, and clearly shows the interaction between frontier technical developments, not only in replacement, but also in the application of the reduction R. Other key method developments were related to introduction of new *in vitro* and *in silico* methods for screening in a tiered approach during the “design-make-test-analyse cycle” and lead optimization procedures, and prior to *in vivo* testing. Introduction of *in vitro* and *in silico* methods is often categorized as an example of replacement rather than reduction. However, the present study gives examples of how non animal methods can be used to guide the subsequent *in vivo* studies in a reduction direction. Based on these observations, it is therefore recommended to coordinate the development of new *in vitro* methods with the workings of the *in vivo* departments, and to further build on a paradigm where *in silico-, in vitro-* and *in vivo*-methods all hold the potential for applying the reduction R.

In addition to study design and method improvements, eleven projects used tactical coordination as a mean for reduction (Table 3). In comparison to the other two strategies, the quantifiable impact by better project coordination was more uncertain. Two projects related to the combination of studies were not possible to evaluate in quantitative terms within the short time-frame of this study, since the number of studies vary substantially between years. However, combining tests related to different departments and minimizing the purchase of “extra” animals reduced the number of rats in quantifiably terms. Finally, four projects were related to new approaches for cooperation within the unit, such as establishment of bio-banks and systematic search for biomarkers. Here it is estimated that savings would be another 5–10% (250–500) annually. The active coordination within a research unit represents a major in-road in the application of 3R principles into the central risk assessment strategies of the pharmaceutical industry, particularly in the area of regulatory, repeated dose, systemic toxicity testing. A pre-clinical bio-bank may for example hold the potential of new research on biomarkers and validation of new test methods.

The present data shows a two-fold increase of pivotal safety assessment support to the drug projects at the company, whilst the number of rats used remained at unchanged levels (Figure 2). The research unit obtained more information from the same number of animal used, which demonstrates that the systematic 3R work at the research unit has resulted in a reduction, according to the definition. For example, toxicokinetic data was achieved from individual animals rather than from satellite animals. The highest reduction was achieved in the use of rats, the most common laboratory animal at the facility (Figure 3). Several projects were also applicable for other species and the reduction was also significant for mice, rabbits and dogs. These results indicate that the identified strategies for reduction via new study designs, methods and coordination have an inter-species relevance.

### Process management

There is a considerable pressure from society on the area of biomedical research to apply 3R principles to research involving animal experimentation. Central to this is the reduction in animal use which could have a major impact in multiple areas of research. Although officially embraced in various legislations and guidelines, the discrepancy between society's 3Rs vision and practices is well illustrated in public surveys [22]–[24]. There is a documented fear among researchers that applying the reduction R would compromise the informative value of the study. In highly regulated areas, like the pharmaceutical industry, statements as *“without changing the OECD guidelines it is almost impossible to reduce the number of animals in regulatory toxicity studies”* are not uncommon. The importance of fostering a culture of communication and collaboration in the use of animals in biomedical research was explored by tracking the project to the initiating department. The initiative and implementation of the reduction projects was organized from all departments at the lab (Figure 4), indicating that the 3R thinking and practice were integrated in everyday work at all levels in the studied organization. In more than one out of four projects (10/36), the initiative and project steering was cross departmental, showing that the 3R culture had effectively infiltered the whole organization. Here, management support, combined with technical advances, e.g. new assays/methods, increased level of knowledge and development of computer programs, were all key factors enabling the staff to go from idea to implementation. In terms of key management activities, the establishment of cross-departmental teams for 3R-overview and support of the ethical applications and a new strategy for internal and external communication related to 3R were vital components in establish the 3R culture. According to the initiators of the different projects, this 3R working culture allowed ideas to be expressed, tested and evaluated considering both excellent science as well as ethical and enduring developments. At an organizational level, the 3R work at the research unit was supported by structured continuous improvement steering with mutual vision, goals and strict follow-ups, according to the Lean Sigma approach. Another important factor to increase 3R awareness was the involvement of all working categories within the organization. Thus, it was essential to include animal technicians in the research process, to give them more responsibilities in experimental procedures and authority to report observations and research data. The increased motivation and confidence of this working category was indicated by the highest proportional involvement in cross-departmental 3R projects at the unit (Figure 4).

It is evident from this study that the organizational culture, defined as a pattern of basic assumptions that help to form a common way for group members to understand and solve problems, is equally or more important than the implementation of the 3R principles into legal regulations and national/international guidelines. This conclusion is also supported by previous work [31]. A lack of organizational 3R culture can be seen as a concern among researchers that application of the 3Rs could risk the scientific quality and increase costs [22], or that animal pain and distress is inversely related to reduction of animals [32]. In the present study, we show that these concerns are not valid. The quality of data was assured by internal validation and cost effectiveness increased substantially. Even though reduction is not accompanied to refinement *per se*, the present study shows examples of synergy between the two Rs, noted in a project related to decreased group size due to high pregnancy rate, as a result of high quality animal welfare. In addition, the new micro-sampling technique resulted in a reduced number of utilized rats and mice, but also in substantially reduced discomfort at blood sampling as compared to the technique for larger volumes samples [33], [34]. Also, the micro-sampling resulted in higher scientific quality with simultaneous evaluation of toxicity and toxicokinetic information.

### From pharmaceutical industry and beyond

Several arguments support that the strategies identified in the present study might be transferred outside the context of a pharmacological industry. Firstly, the constant pressure to minimize the use of laboratory animals is considerable throughout the whole biomedical sector, and for the safety assessment area in specific. Subsequently, the demands from legislations such as REACH and the new EU-directive for laboratory animals are related to other chemical products and any use of animals [4]. There is also a need to further advances and harmonisation of the 3Rs in different regulatory sectors [35]. In addition, the initiatives to implement new methods (e.g. the successful implementation of micro-sampling in this study) can be used in many different types of investigative and regulatory experiments. Methodological interventions, as compared to improved study design and coordination, are also most likely to be transferrable into other research areas within Life Science. A similar retrospective survey was previously performed in a medium-sized pharmaceutical company [36], where the ratio of animal use per compound synthetized showed a decrease with 80% as measured over 12 years (1991–2002). This reduction coincided with introduction of various *in vitro* screening tests, indicating a causal relation. No further analyses on how the reduction was achieved in more detail were, however, presented. Further, systematic coordination can be applied in any organization. Despite this, it might be more difficult to implement these strategies in scattered organizations, such as academia or sub-contracted contract research organizations (CROs). Also, the resources to validate innovative methods and new study designs is often limited in non-industrial organizations in which the economic efficiency derived through reduction cannot be directly exploited. Previous examples of successful 3R initiatives in toxicity testing have been variously reported from different areas of endeavor, but have often been very focused on particular tests and use areas. It has, for example, been shown that regulatory requirements of acute toxicity testing of pharmaceuticals can be replaced as acceptable supporting information can be achieved from single high dose toxicity studies [37]. One of the most influential general reports on this subject is the OECD list of thirty 3R-improved or up-dated tests for short- and long-term toxicity testing, complete with description of the 3R relevance and changes made (Table available for download at http://www.oecd.org/env/ehs/testing/44146476.pdf). For example, the first alternative to the conventional acute toxicity test (TG401) is the revised version of TG420, adopted in 2001. This is an example of a new study design with fixed doses and testing in one sex only (usually females). Another example of study design improvement is the recent revision of the OECD guideline for bioaccumulation tests in fish, resulting in a reduction with approximately 30% [38]. In summary, the international development of validated and recognized methods and study designs is important, but suffers from the time-consuming process before acceptance within the OECD framework. Other reports of reduction are sporadic in the literature and do not bear any concerted efforts across a whole safety testing strategy. In the present study, all 3R-projects were devised, internally validated and approved before implementation. To ensure the scientific quality of the data generated after the implementation of the reduction, a proper validation must be performed concerning prediction/translation, statistical power and working situation for personnel and animals etc. As validation is a crucial step, the European Union Reference Laboratory for Alternatives to Animal Testing (EURL-ECVAM) has organized a network of validation laboratories [39]. This is certainly an important initiative to counterstrike the lack of international resources for validation. However, in the present study we are able to show the importance of internal validation and rapid implementation within an area framed with strict regulations (i.e. pharmaceutical industry). In fact, four of the projects related to improved study design, (combining studies and changing dosing routine; No 2, 3, 5 and 6 in table 1) resulted from reinterpretation of regulatory guidelines, but with sustained delivery of scientific information.

In conclusion, it is evident from this study that substantial reductions in animal use in research can be achieved by different strategies, including improved study design, method development and project coordination. Major animal savings were shown in both regulatory and investigative safety studies. If a similar (i.e. 53%) reduction had been achieved simultaneously within the twelve largest pharmaceutical companies, the equivalent reduction world-wide would be about 150,000 rats annually, assuming that the use of laboratory animals is proportional to their respective economic turnovers. The work points at the importance of a strong 3R culture, within the organization, with scientific engagement, collaboration and a responsive management being vital components. Based on the results from this study we recommend a strong commitment in leadership for the 3R to be translated into cross-department and inter-profession involvement in projects for innovation, validation and implementation. We also observe clear examples of synergies between all the three Rs and conclude that *in silico-, in vitro-* and *in vivo*-methods all hold the potential for applying the reduction R and should be consequently coordinated at a strategic level.
